# Biodegradable Zinc Oxide Nanoparticles Doped with Iron as Carriers of Exogenous Iron in the Living Organism

**DOI:** 10.3390/ph14090859

**Published:** 2021-08-27

**Authors:** Paula Kiełbik, Aneta Jończy, Jarosław Kaszewski, Mikołaj Gralak, Julita Rosowska, Rafał Sapierzyński, Bartłomiej Witkowski, Łukasz Wachnicki, Krystyna Lawniczak-Jablonska, Piotr Kuzmiuk, Paweł Lipiński, Marek Godlewski, Michał Marek Godlewski

**Affiliations:** 1 Institute of Veterinary Medicine, Department of Physiological Sciences, Warsaw University of Life Sciences, 02-787 Warsaw, Poland; mikolaj_gralak@sggw.edu.pl (M.G.); michal_godlewski@sggw.edu.pl (M.M.G.); 2 Institute of Genetics and Animal Biotechnology, Department of Molecular Biology, Polish Academy of Sciences (PAS), 05-552 Jastrzębiec, Poland; a.jonczy@igbzpan.pl (A.J.); p.lipinski@ighz.pl (P.L.); 3 Institute of Physics, Polish Academy of Sciences (PAS), 02-668 Warsaw, Poland; kaszewski@ifpan.edu.pl (J.K.); rosowska@ifpan.edu.pl (J.R.); bwitkow@ifpan.edu.pl (B.W.); lwachn@ifpan.edu.pl (Ł.W.); jablo@ifpan.edu.pl (K.L.-J.); kuzmiuk@ifpan.edu.pl (P.K.); godlew@ifpan.edu.pl (M.G.); 4 Division of Pathology, Institute of Veterinary Medicine, Department of Pathology and Veterinary Diagnostics, Warsaw University of Life Sciences, 02-776 Warsaw, Poland; rafal_sapierzynski@sggw.edu.pl

**Keywords:** iron oxide nanoparticles, iron deficiency, oral iron, nanotechnology, iron delivery, hepcidin, anemia

## Abstract

Iron plays an important role in various crucial processes in the body and its deficiency is considered currently as a serious health problem. Thus, iron supplementation strategies for both humans and animals need to be effective and safe. According to our previous studies, zinc-based nanoparticles provide safe, biodegradable, fast and efficient transport system of orally given substances to the tissues. In the current manuscript we present results of a study aimed at investigation of the ZnO nanoparticle-based Fe supplementation system (average size 100 × 250 nm). Nanostructures were orally (gavage) administered to adult mice. Animals were sacrificed at different time points with collection of blood and internal organs for analyses (tissue iron concentration, hepatic level of hepcidin, blood parameters, liver and spleen levels of ferritin, histopathology). Initial experiment was performed to compare the biological effect of doping type (Fe^3+^ doping vs. a mixture of Fe^3+^ and Fe^2+^). Then, the effect of acute/chronic exposure models was determined. The increase in ferritin, along with improved, crucial hematological parameters and lack of the influence on hepcidin expression indicated the chronic application of Fe^3+,2+^ doped ZnO nanostructures to be the most effective among tested.

## 1. Introduction

Iron deficiency has a considerable impact on human health and the economy. According to the World Health Organization iron deficiency is the most common nutritional deficiency (approximately one third of the human population) and the most common cause of anemia [1,2]. Iron plays an important role in various crucial processes in the body [3,4] and its deficiency in pregnant women might cause health problems of neonates [5]. Moreover, proper iron body reserves are important for both the physical and neuropsychological development of children [6,7]. For therapeutic purposes, iron is usually delivered to the living organism through oral, intramuscular or intravenous route. Oral application seems to be the most natural, since it involves various pathways of iron absorption/transport from the gastrointestinal tract (GI). Dietary iron is found in two forms: non-heme (Fe^3+^) and heme (Fe^2+^) iron. Heme iron is better absorbed in the GI than Fe^3+^ ions [8]. Fe^3+^ ions might also contribute to toxic effects as they must be converted into chemically active Fe^2+^ during membrane passage. Physiological mechanisms of digestive system usually prevent creation of toxic reactive oxygen species (ROS) caused by presence of free, unbound heme iron ions (Fe^2+^). The key factors limiting the efficiency of oral iron administration are related with low bioavailability of tested iron supplements [9]. Reports indicate that oral iron supplementation also affects gut microbiota [10]. Developing a new, more efficient, safe and simple iron supplementation strategy in human population is therefore important.

Likewise, iron deficiency is a common, widely recognized condition in neonatal piglets [9,11]. In order to prevent anemia, an intramuscular administration of iron-dextran is usually given [12,13,14]. However, some significant disadvantages of this strategy have been identified. Poor iron injection techniques can cause considerable destruction to the muscles or spread various diseases in the herd [15]. Furthermore, parenteral intake of high amounts of exogenous iron can lead to excessive oxidative stress [16,17], mostly related with ROS creation through Fenton reaction [18]. Intramuscular injection of iron also contributes to overwhelming the natural system controlling the amount of circulating iron, based on hepcidin [19,20,21,22,23].

Nowadays, nanostructures have become greatly popular among scientists as innovative tools for various biomedical purposes [24,25,26,27]. Nanoparticles reveal completely different features compared to substances in bulk or their ionic equivalents, including different transcellular transport systems. It has been suggested to utilize the alternative pathways of nanoparticle absorption in the gut to improve bioavailability of deficient substances to the living organism [28]. Iron nanoparticles have been widely studied because of their magnetic properties. Thus, they have been used in biomedical studies, especially as drug delivery and for magnetic resonance imaging [29,30,31,32]. However, the use of iron-doped nanoparticles as potential carriers of exogenous iron to the living organism is novelty. According to our previous studies, zinc-based nanoparticles provide a safe, biodegradable, fast and very efficient transport system for orally given substances to key organs [33,34,35,36]. Promising results of mentioned studies prompted the start of research on zinc-based nanoparticles doped with iron. Our preliminary studies in mice showed deposition of iron in the key tissues involved in iron metabolism. Furthermore, no side effects associated with intra-muscular iron deposition were observed following oral administration of iron-doped nanoparticles [28]. In the current manuscript we present results of comprehensive study aimed at precise investigation of the possibility of utilization of a nanoparticle-based iron supplementation system. From a physiological point of view, the impact of administered iron-doped nanoparticles on blood parameters and the mechanisms related to iron uptake, transport and homeostasis were studied.

## 2. Results

### 2.1. Characterization of Nanoparticles

**Figure 1:**
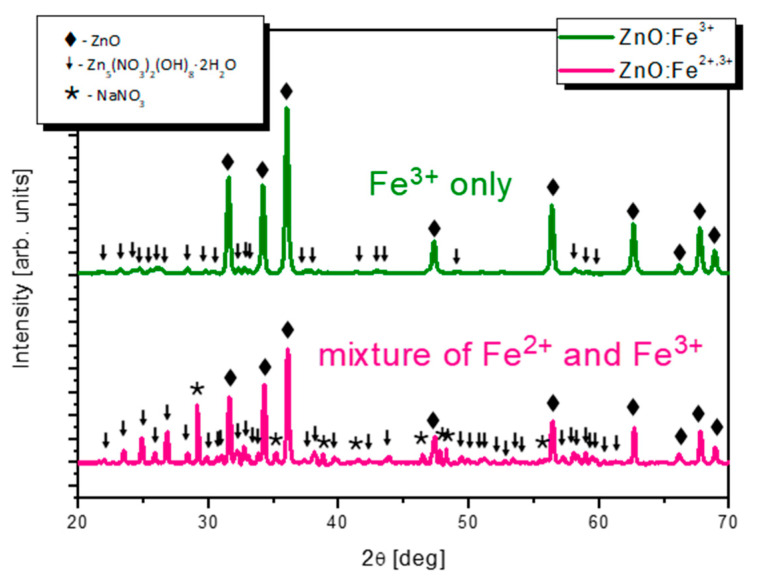
X-ray diffraction patterns of ZnO:Fe^3+^ nanoparticles (upper) and ZnO:Fe^3+^ enriched with iron 2+ ions (lower pattern). Marked by the patterns: ◆—wurtzite type ZnO phase (PDF no. 79-0208), ↓—Zn_5_(NO_3_)_2_(OH)_8_∙2H_2_O (PDF no. 24–1460), 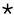
—NaNO_3_ (PDF no. 36-1474).

X-ray diffraction patterns of nitrate(V) (ZnO:Fe^3+^) and acetate (ZnO:Fe^3+,2+^) samples are shown in Figure 1. Both samples contain wurtzite type zinc oxide phase (PDF no. 79–0208). In ZnO:Fe^3+^ only sample wurtzite phase dominates, however traces of Zn_5_(NO_3_)_2_(OH)_8_∙2H_2_O (PDF no. 24-1460) are also present. On the other hand, synthesis using iron(II) acetate results in a mixture of ZnO, Zn_5_(NO_3_)_2_(OH)_8_∙2H_2_O and NaNO_3_ (PDF no. 36-1474, Figure 1). Zn_5_(NO_3_)_2_(OH)_8_∙2H_2_O is poorly soluble compound seemingly indicating that certain quantity of starting zinc hydroxide has not reacted. More zinc hydroxide nitrate reacts when nitrates(V) are starting compounds, less reacts in synthesis with the use of acetate. Zn_5_(NO_3_)_2_(OH)_8_∙2H_2_O and NaNO_3_ (nitratine) phases appear suggesting a heavy contamination of Fe^2+^ sample.

**Figure 2:**
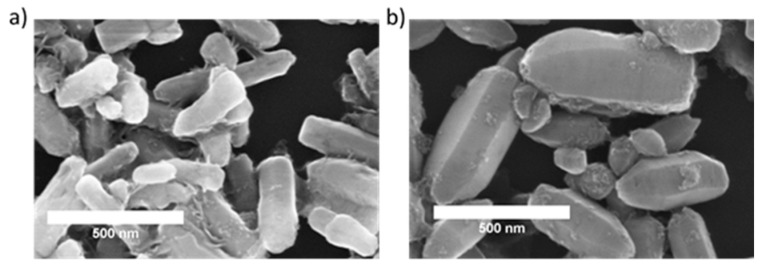
Scanning electron microscopy images of ZnO:Fe^3+^ (**a**) and ZnO:Fe^3+,2+^ (**b**) nanoparticle structures.

Scanning electron microscopy (SEM) images are shown in Figure 2. Both samples of ZnO consist of elongated crystals shaped as hexagonal prisms, slightly narrowed at the ends. In the sample containing Fe^3+^ only, crystals are: 200–500 nm in length and ca. hundred nanometers wide. Sample with mixture of Fe^2+^ and Fe^3+^ contains crystals divided into two fractions: hundreds of nanometers long and 200–300 nm wide prisms and smaller, rounder crystals below 100 nm in size.

The elemental composition measured by energy-dispersive X-ray spectroscopy (EDX) has shown (Table 1), that larger nanoparticles observed in ZnO:Fe^3+,2+^ exhibit a lower metals to oxygen rate (0.33 versus 0.71 in ZnO:Fe^3+^) and nitrogen occurs with higher content in this sample (11.6 at% versus 5.1 at%). This confirms higher concentration of Zn_5_(NO_3_)_2_(OH)_8_∙2H_2_O and sodium nitrate(V) phases observed in XRD measurements (Figure 1).

The elemental composition measured by X-ray photoelectron spectroscopy (XPS) has shown (Table 2) similar as in EDS the excess of oxygen in relation to zinc in single Fe valence sample (0.64). Nevertheless, in sample containing mixed valence Fe ions this ration is closer to stoichiometric (0.80) opposite to EDX (0.33) indicating that surplus of oxygen is inside the observed crystallites due to surface sensitivity of XPS. Moreover, this is confirmed by small N content at the surface of this sample, despite the larger content of hydroxide phase (Zn_5_(NO_3_)_2_(OH)_8_∙2H_2_O), which contains 3.2 oxygen atoms in stoichiometric unit per 1 Zn atom. Therefore, from comparison of EDX and XPS measurements we can state that detected hydroxide phase is located in the bulk of the crystallites in these samples.

Concentration of iron in all cases is close to 1 atomic %, taking into account decrease of accuracy of used techniques for low concentration of element.

**Figure 3:**
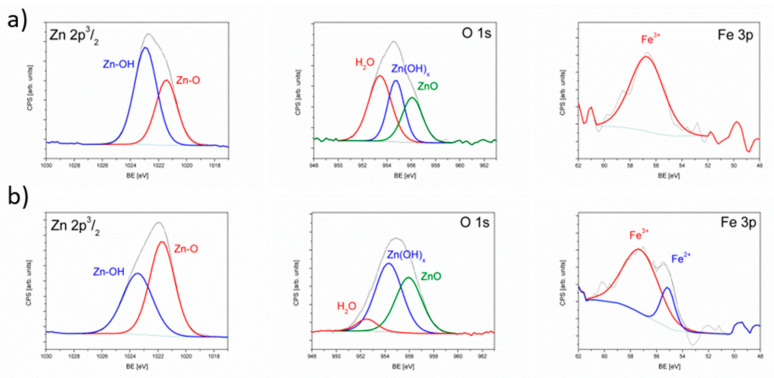
XPS analysis of Zn 2p, O 1s and Fe 3p lines in ZnO:Fe^3+^ sample (**a**) and ZnO:Fe^3+,2+^ sample (**b**).

XPS chemical analysis of the Zn 2p 3/2 and Fe 3p spin-orbit doublet, and O 1s orbital is shown in Figure 3 for both samples. The good envelope can be obtained introducing for Zn 2p3/2 two components at the binding energy (BE): 1021.4 eV and 1022.9 eV. Component at 1022.9 eV is assigned to Zn bound with -OH groups and 1021.4 to Zn bound to O ions in ZnO crystals [37]. Hydroxyl bound Zn is more abundant in Fe^3+^ sample (61%) than in Fe^2+^/Fe^3+^ sample (44%), confirming again that phase containing -OH ions is located in the bulk of crystallites. Moreover, the full width at half maximum (FWHM) of this component is broader in mixed Fe valence sample what is usually correlated with larger disorder in the related compound. The O 1s line exhibits three components at BE: 530.5 eV, 531.9 eV and 533.2 eV which correspond to oxygen bound to Zn in ZnO, oxygen in zinc hydroxide species and crystallization water, respectively. In Fe^3+^ only sample, H_2_O component (533.1 eV) is more intensive, indicating presence of more crystallization water at the surface of this sample, however, ZnO component is smaller (27%) than in the mixed valence sample (41%). Again, the FWHM of the components in mixed valence sample are wider, confirming the higher disorder in the position of oxygen. Due to the presence of the Zn Auger lines between spin orbit Fe 2p lines, the analysis of Fe 3p line was performed. Knowing that the 2p line for Fe^3+^ has BE at 57 eV and for Fe^2+^ ions at 55 eV [37], it was confirmed in the mixed valence sample the presence of 20% of Fe^2+^ ions, whereas for single valence sample the single line was sufficient to get good envelope of the measured Fe 3p spectra.

**Figure 4:**
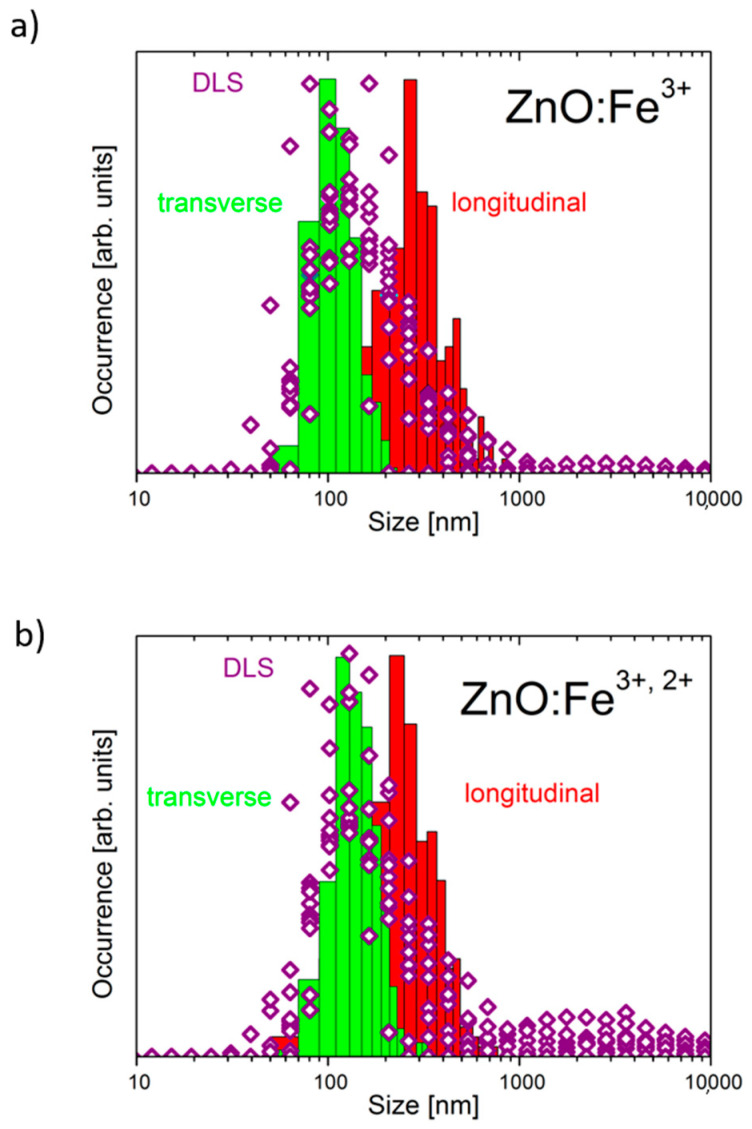
Nanoparticle size distribution as measured using dynamic light scattering (purple diamonds) and scanning electron microscopy techniques (green and red bars, respectively for transverse and longitudinal measurements). (**a**) ZnO:Fe^3+^, (**b**) ZnO:Fe^3+,2+^.

Nanoparticle size distribution is shown in Figure 4. Histograms show distribution of sizes according to SEM images and were completed for transverse (green bars) and longitudinal (red bars) dimensions of visible objects. Purple diamonds show distribution obtained from water suspension of samples using dynamic light scattering (DLS) method. In ZnO:Fe^3+^ sample maxima of transverse and longitudinal dimensions of measured objects are 99 nm and 271 nm, respectively. In ZnO:Fe^3+,2+^ maxima are located 119 nm and 231 nm, therefore similar values suggest closer to circular shapes of nanostructures. DLS results reveal that in both cases (samples Fe^3+^ only and Fe^3+,2+^) size distributions are log-normal. Additionally, there is a small population of structures (larger in ZnO:Fe^3+,2+^ sample) sized from 1 µm to 10 µm, likely resulting from agglomeration of crystals in suspension (Figure 4). In histograms prepared using SEM technique, large population is not visible, which is related to the sample preparation for measurements (droplet of water suspension placed on silicon wafer). Distributions prepared by both methods are strongly overlapping in the region of 50–1000 nm.

**Figure 5:**
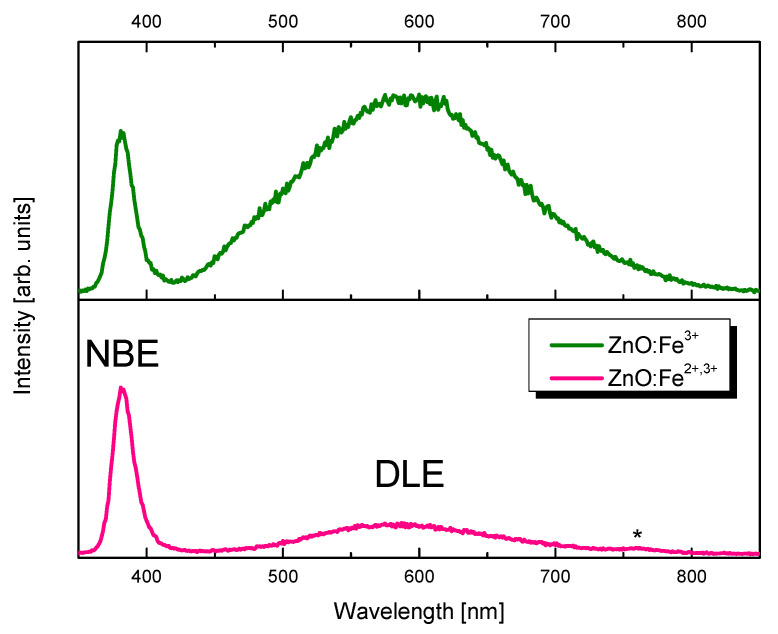
Cathodoluminescence of ZnO:Fe^3+^ (upper) and ZnO:Fe^3+,2+^ (lower) nanoparticles. Asterisk shows measurement artifact. NBE—near bandedge emission, DLE—deep level emission.

**Figure 6:**
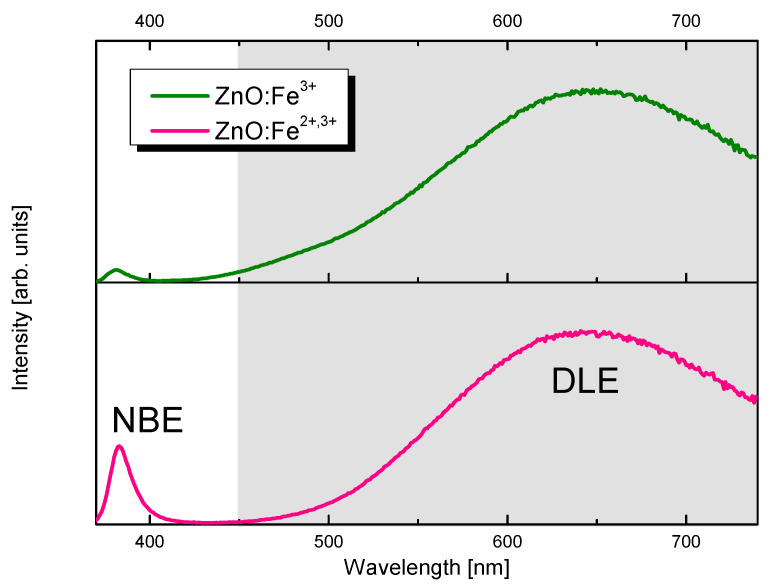
Photoluminescence spectra (λ_exc_ = 300 nm) of ZnO:Fe^3+^ (upper) and ZnO:Fe^3+,2+^ (lower) samples.

To evaluate quantity of defects in ZnO:Fe samples, $\frac{I_{DLE}}{I_{NBE}}$ ratio (*I_DLE_*—intensity of deep level emission in ZnO, *I_NBE_*—near band edge emission in ZnO) was calculated using integrated area on luminescence spectra in the ranges: NBE 370–450 nm (*I_NBE_*); DLE 450–750 nm (*I_DLE_*). Intensity rate was calculated for cathodoluminescence (Figure 5) and photoluminescence (Figure 6). Cathodoluminescence spectra of prepared samples are shown in Figure 5. Analysis of deep level emission (DLE) intensity and localization gives information concerning defects [38]. Ratio for sample ZnO:Fe^3+^ is 9.90 and for ZnO:Fe^3+,2+^ is 1.78. It shows a relative intensity of DLE band being ~5.5 times larger in Fe^3+^ only sample and suggests ca. five times higher concentration of defects. Defecting of the structure in ZnO:Fe sample may result from charge difference between Zn^2+^ and Fe^3+^ ions. Furthermore, comparison of ionic radii of Zn^2+^ and Fe^2+^ in tetrahedral coordination (0.6 Å and 0.63 Å, respectively [39]), explains, that presence of Fe^2+^ in the ZnO:Fe^3+,2+^ sample structure allows preferable substitution of Fe^2+^ into Zn^2+^ lattice sites. Thus Fe^2+^ induces fewer defects in the nanocrystals than presence of Fe^3+^, with ionic radius of 0.49 Å. This trend is preserved in the photoluminescence spectroscopy. In the sample ZnO:Fe^3+^ *I_DLE_* to *I_NBE_* ratio is 149.28 and in the ZnO:Fe^3+,2+^ sample is 24.60 meaning defect-related band is ca. six times stronger in ZnO:Fe^3+^ only sample.

Measurements in suspension indicated that Zeta potential of ZnO:Fe^3+^ sample was +66.056 mV and ZnO:Fe^3+,2+^ sample was +70.946 mV. This shows that nanoparticles are positively charged and very stable in aqueous solution.

### 2.2. Selection of ZnO Nanoparticles Doping-Fe^3+^ or Fe^3+,2+^

**Figure 7:**
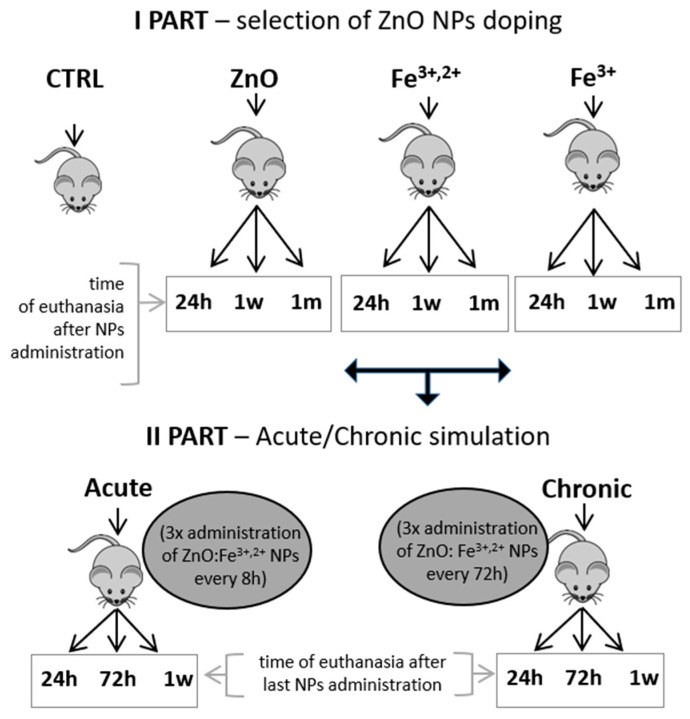
Schematics of animal study design (NPs—nanoparticles; 1 w—1 week; 1 m—1 month).

All animal studies were performed following Polish and European guidelines for animal experiments, Local Ethical Committee agreement No WAW2/59/2017. Initial part of the animal study was performed to compare the biological effect of both ZnO nanoparticle doping (single Fe^3+^ iron or a mixture of Fe^3+^ and Fe^2+^). Mice were divided into following groups: control group CTRL, ZnO (ZnO nanoparticle suspension in reverse osmosis (RO) water); Fe^3+,2+^ (ZnO nanoparticles doped with Fe^3+,2+^); and Fe^3+^ (ZnO nanoparticles doped with Fe^3+^). Nanoparticles were administered to mice by gavage and after 24 h, one week or one month, animals were sacrificed (for details see Material and Methods section). Blood and internal tissues were collected for analyses (Figure 7).

**Figure 8:**
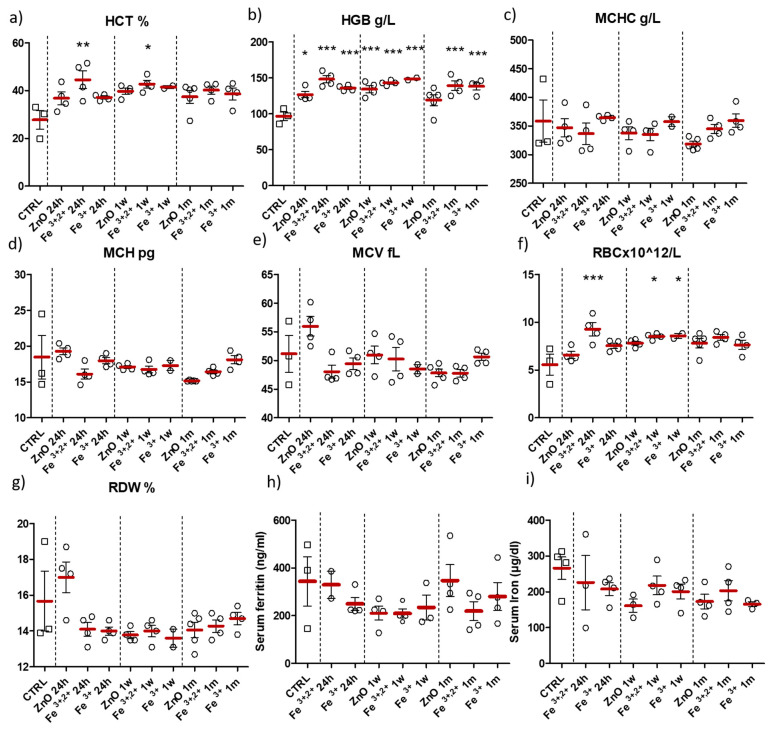
Hematological parameters, serum ferritin and iron levels in analyzed blood samples after intra-gastric administration (gavage) of ZnO, ZnO:Fe^3+^ or ZnO:Fe^3+,2+^ nanoparticle suspension in RO water vs. the control group (CTRL). Animals were sacrificed following 24 h, 1 week (1 w) or 1 month (1 m) after nanoparticle application. Hematocrit-HCT (**a**), hemoglobin concentration-HGB (**b**), mean corpuscular hemoglobin concentration-MCHC (**c**), mean corpuscular hemoglobin-MCH (**d**), mean corpuscular volume-MCV (**e**), erythrocyte count-RBC (**f**), red cell distribution width-RDW (**g**), serum ferritin (**h**) and iron (**i**) levels were measured in mice blood. Data presented as mean (±SEM) for control (*n* = 3 for (**a**–**h**)/*n* = 4 for (**i**)) vs. experimental groups (ZnO 24 h *n* = 4; Fe^3+,2+^ 24 h *n* = 4; Fe^3+^ 24 h *n* = 4; ZnO 1 w *n* = 4; Fe^3+,2+^ 1 w *n* = 4; Fe^3+^ 1 w *n* = 2–4; ZnO 1 m *n* = 5; Fe^3+,2+^ 1 m *n* = 4; Fe^3+^ 1 m *n* = 4). Statistically significant differences of * *p* ≤ 0.05, ** *p* ≤ 0.01, and *** *p* ≤ 0.001 were accordingly indicated. Range presented as squares for CTRL and circles for experimental groups.

Blood collected from all groups were analyzed for hematological parameters, level of serum ferritin and serum iron content (Figure 8). Erythrocyte count and hematocrit significantly increased in two Fe^3+,2+^ groups at 24 h and one week after nanoparticle application (Figure 8a,f). In case of the red blood cell count (RBC), an increase was also detected in group ZnO:Fe^3+^ one week following administration (Figure 8f). The level of hemoglobin was significantly increased in almost all experimental groups (except the ZnO nanoparticle group one month after application) (Figure 8b). Mean corpuscular hemoglobin (MCH), mean corpuscular hemoglobin concentration (MCHC), mean corpuscular volume (MCV) and red blood cell distribution width (RDW) did not significantly differ between experimental groups and control (Figure 8c–e,g). Both serum iron and ferritin levels did not show any significant changes within experiment (Figure 8h,i).

**Figure 9:**
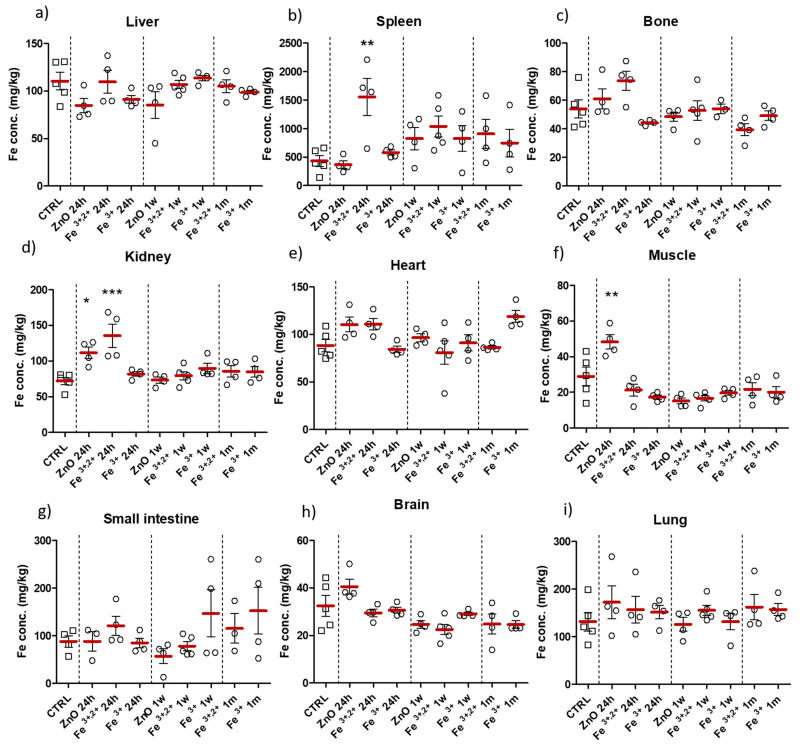
Tissue accumulation of iron in control group (CTRL) and after intra-gastric administration of ZnO, ZnO:Fe^3+^ or ZnO:Fe^3+,2+^ nanoparticles. Data represent mean level of iron concentration in tested organs (mg/kg of tissue), at different time following nanoparticle application (24 h, 1 w or 1 m). Data presented as mean (±SEM) for control (*n* = 5) vs. experimental groups (ZnO 24 h *n* = 4; Fe^3+,2+^ 24 h *n* = 4; Fe^3+^ 24 h *n* = 4; ZnO 1 w *n* = 4; Fe^3+,2+^ 1 w *n* = 5; Fe^3+^ 1 w *n* = 4; Fe^3+,2+^ 1 m *n* = 4; Fe^3+^ 1 m *n* = 4). Statistically significant differences of * *p* ≤ 0.05, ** *p* ≤ 0.01, and *** *p* ≤ 0.001 were accordingly indicated. Range presented as squares for CTRL and circles for experimental groups.

Quantitative evaluation of iron concentration in collected tissue samples were determined with flame Atomic Absorption Spectrometry (AAS). Established iron concentration in the collected tissues remained mainly unchanged (comparing to control group) or without meaningful tendency (Figure 9). Significant increase in iron level, in comparison to control, was observed in the spleen, bone (trend) and kidney 24 h after application of ZnO:Fe^3+,2+^ nanoparticles (Figure 9b–d). Likewise, 24 h following administration of ZnO nanoparticles, significantly elevated iron level was observed in the muscle and brain (trend) (Figure 9f,h).

**Figure 10:**
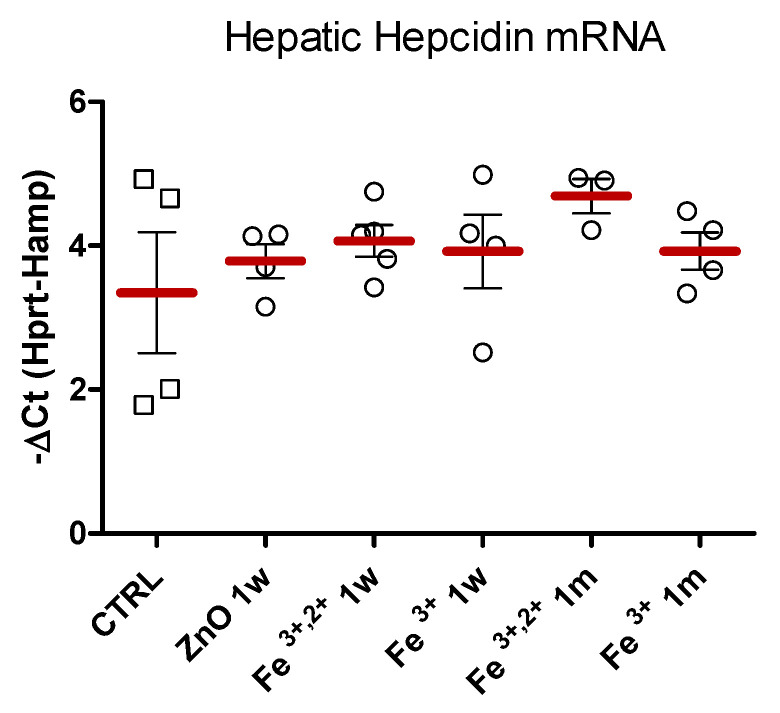
Hepatic hepcidin mRNA levels measured with RT-PCR method and normalized to transcripts encoding hypoxanthine-guanine phosphoribosyltransferase genes (Hprt). Results obtained at different time points (1 w and 1 m) after intra-gastric administration of ZnO, ZnO:Fe^3+,2+^ or ZnO:Fe^3+^ nanoparticles versus the control group (CTRL). Data presented as mean (±SEM) for control (*n* = 4) vs. experimental groups (ZnO 1 w *n* = 4; Fe^3+,2+^ 1 w *n* = 4; Fe^3+^ 1 w *n* = 4; Fe^3+,2+^ 1 m *n* = 4; Fe^3+^ 1 m *n* = 4). Range presented as squares for CTRL and circles for experimental groups.

The level of hepatic hepcidin mRNA (Figure 10), analyzed with RT-PCR method showed no statistically significant changes between experimental and control groups.

**Figure 11:**
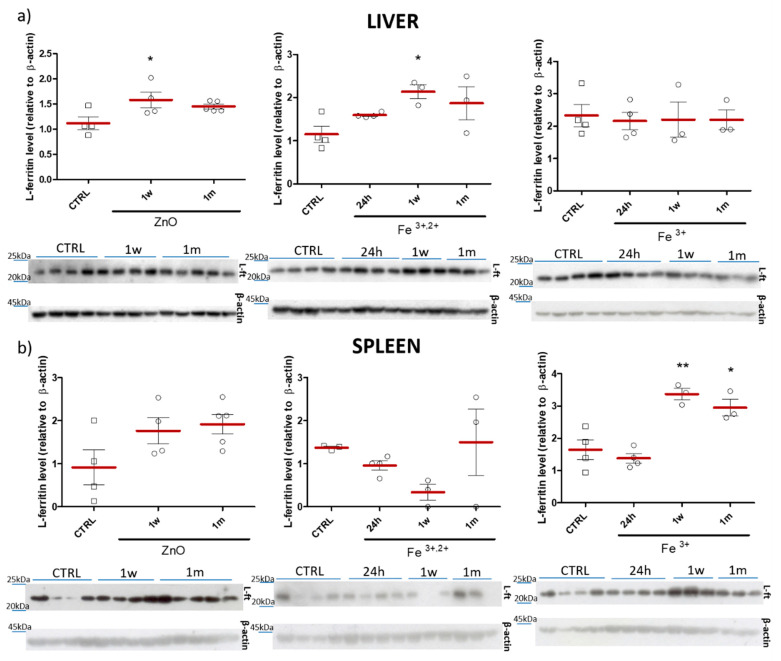
Western blot densitometric analyses of the levels of L-ferritin subunit in the liver (**a**) and spleen (**b**). Representative immunoblots are shown below plots. Results obtained 24 h, 1 w or 1 m after intra-gastric administration of ZnO, ZnO:Fe^3+^ or ZnO:Fe^3+,2+^ nanoparticles vs. the control group (CTRL). Data presented as mean (±SEM) for control (*n* = 4) vs. experimental groups (ZnO 24 h *n* = 4; ZnO 1 w *n* = 4; ZnO 1 m *n* = 5; Fe^3+,2+^ 24 h *n* = 4; Fe^3+,2+^ 1 w *n* = 3; Fe^3+,2+^ 1 m *n* = 3; Fe^3+^ 24 h *n* = 4; Fe^3+^ 1 w *n* = 3; Fe^3+^ 1 m *n* = 3). Statistically significant differences of * *p* ≤ 0.05 and ** *p* ≤ 0.01 were accordingly indicated. Range presented as squares for CTRL and circles for experimental groups.

Immunoblot analyses revealed a significant increase (vs. control) of the liver L-ferritin subunit levels in mice 1 w following administration of ZnO and ZnO:Fe^3+,2+^ nanoparticles (Figure 11a). On the other hand, in the spleen, a significant increase of the liver L-ferritin subunit was observed only 1 w and 1 m after application of ZnO:Fe^3+^ (Figure 11b).

### 2.3. Effect of Acute/Chronic Exposure to ZnO Nanoparticles Doped with Fe^3+,2+^

**Figure 12:**
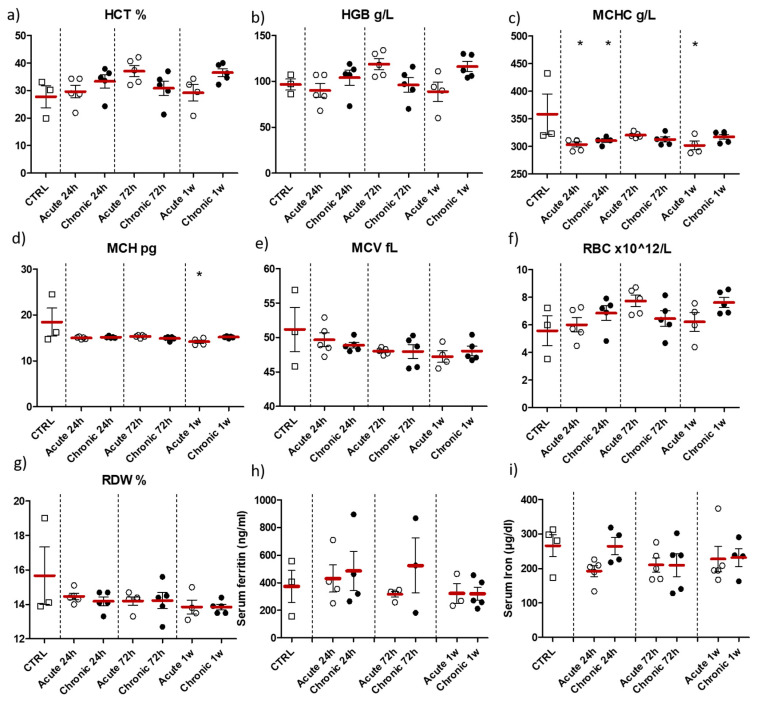
Hematological parameters, serum ferritin and iron levels in the blood samples collected from animals sacrificed following multiple intra-gastric administration of ZnO:Fe^3+,2+^ nanoparticles in acute/chronic simulation. Hematocrit—HCT (**a**), hemoglobin concentration—HGB (**b**), mean corpuscular hemoglobin concentration—MCHC (**c**), mean corpuscular hemoglobin—MCH (**d**), mean corpuscular volume—MCV (**e**), erythrocyte count—RBC (**f**), red cell distribution width—RDW (**g**), serum ferritin (**h**) and iron (**i**) levels were measured at different time-points (24 h, 72 h or 1 w) and plotted against the control group (CTRL). Data presented as mean (±SEM) for control (*n* = 3 for (**a**–**h**)/*n* = 4 for (**i**)) vs. experimental groups (Acute 24 h *n* = 5; Chronic 24 h *n* = 5; Acute 72 h *n* = 5; Chronic 72 h *n* = 5; Acute 1 w *n* = 5; Chronic 1 w *n* = 5). Statistically significant differences of * *p* ≤ 0.05 were accordingly indicated. Range presented as squares for CTRL, open circles for acute model and filled circles for chronic model in experimental groups.

ZnO nanoparticles doped with Fe^3+,2+^ were selected for further simulation of acute/chronic exposure (multiple application of nanoparticles at different time-intervals) based on the results of first part of the study. Animals were euthanized at 24 h, 72 h or 1 w after last nanoparticle application (see Figure 7). Blood (hematological) parameters and the level of serum ferritin and iron of tested animals are shown on Figure 12. A similar increase in red blood cells count (RBC), hemoglobin concentration (HGB) and hematocrit (HCT) was observed 72 h after acute and 1 w after chronic application of ZnO:Fe^3+,2+^ nanoparticles (trend) (Figure 12a,b,f). Mean corpuscular hemoglobin (MCH) and mean corpuscular hemoglobin concentration (MCHC) parameters decreased in all experimental groups, however statistically significant changes were observed only at 24 h and 1 w in the acute application model (when compared to control) (Figure 12c,d). No significant changes were observed in other morphological parameters (MCV and RDW) (Figure 12e,g), nor in the serum iron and serum ferritin levels (Figure 12h,i).

**Figure 13:**
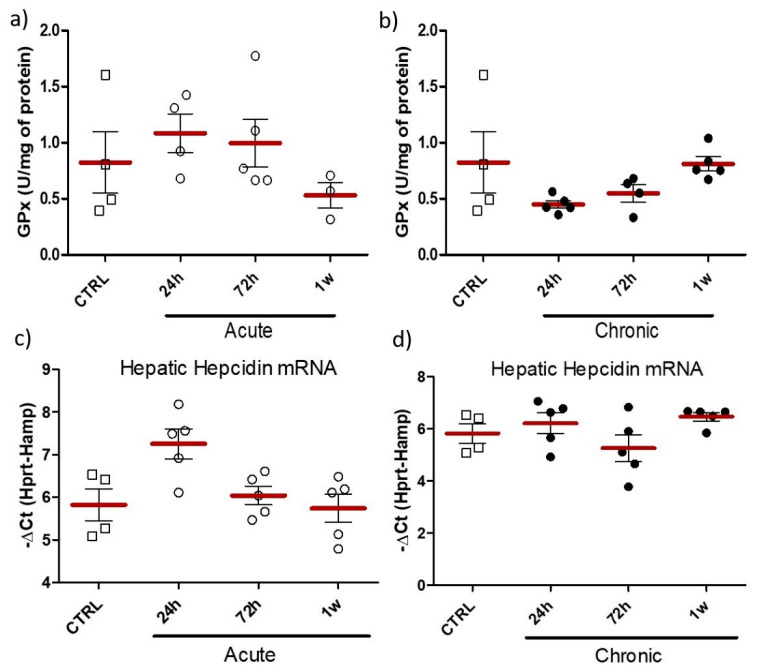
Hepatic activity of glutathione peroxidase in acute (**a**) or chronic simulation (**b**), and mRNA levels of hepatic hepcidin measured with RT-PCR method and normalized to transcripts encoding hypoxanthine-guanine phosphoribosyltransferase genes (Hprt) (**c**,**d**). Results obtained at different time-points (24 h, 72 h or 1 w) following intra-gastric administration of ZnO:Fe^3+,2+^ nanoparticles vs. the control (CTRL). Data presented as mean (±SEM) for control (*n* = 4) vs. experimental groups (Acute 24 h *n* = 5; Chronic 24 h *n* = 5; Acute 72 h *n* = 5; Chronic 72 h *n* = 5; Acute 1 w *n* = 5; Chronic 1 w *n* = 5). Range presented as squares for CTRL, open circles for acute model and filled circles for chronic model in experimental groups.

Activity of hepatic glutathione peroxidase in both acute and chronic simulation did not show any statistically significant changes (Figure 13a,b). However, obtained results indicate increased level of this antioxidant enzyme 24 h and 72 h after administration of nanoparticles in acute simulation (trend) (Figure 13a). No statistically significant changes between experimental and control groups were observed in the level of hepatic hepcidin mRNA, however elevated expression of hepcidin mRNA was observed 24 h following acute application of nanoparticles (trend) (Figure 13c,d).

**Figure 14:**
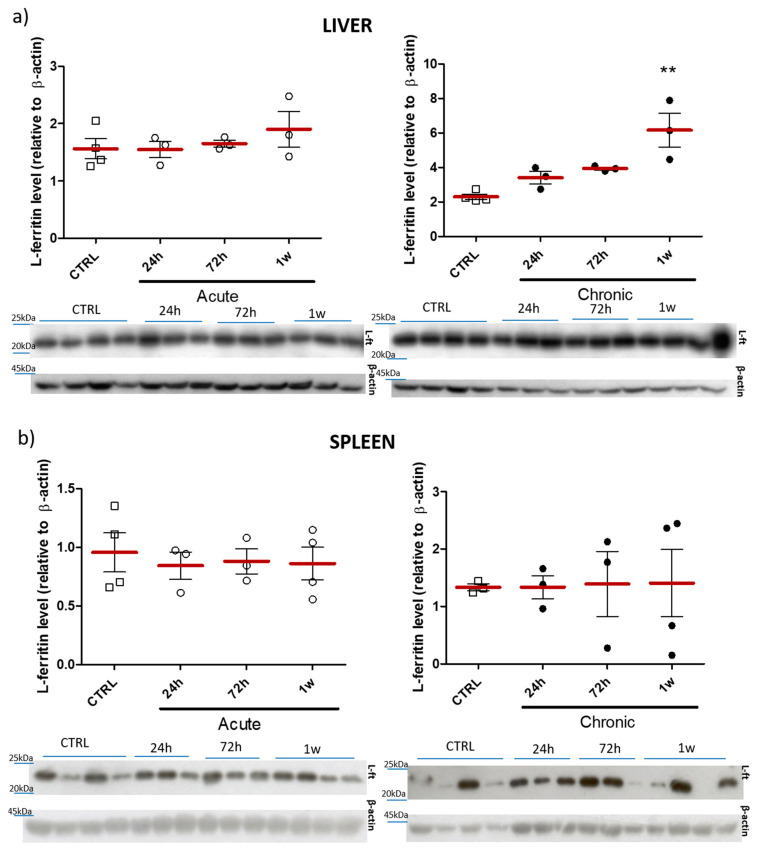
Western blot densitometric analyses of the levels of L-ferritin subunit in the liver (**a**) and spleen (**b**). Representative immunoblots are shown below plots. Results obtained at different time-points (24 h, 72 h or 1 w) after multiple intra-gastric administration of ZnO:Fe^3+,2+^ nanoparticles in acute and chronic simulation modes. Data presented as mean (±SEM) for control (*n* = 4) vs. experimental groups (Acute 24 h *n* = 3; Chronic 24 h *n* = 3; Acute 72 h *n* = 3; Chronic 72 h *n* = 3; Acute 1 w *n* = 3 for (**a**)/*n* = 4 for (**b**); Chronic 1 w *n* = 3 for (**a**)/*n* = 4 for (**b**)). Statistically significant differences of ** *p* ≤ 0.01 were accordingly indicated. Range presented as squares for CTRL, open circles for acute model and filled circles for chronic model in experimental groups.

Immunoblot analysis of the liver L-ferritin subunit, following acute and chronic application of nanoparticles are shown in Figure 14. Presented data indicate a significant increase in level of protein in the liver (*p* ≤ 0.01) at 1 w time-point following simulation of a chronic exposure to nanoparticles (Figure 14a).

### 2.4. Histopathology

Collected tissue samples from all mice (from both parts of the study) were examined by a veterinary pathologist. No differences in histopathology of all analyzed tissues were detected, with exemption of several lung cross-sections where multifocal and mild atelectasis were observed. Since those findings were equally shared between mice from control and experimental groups, we did not consider them as nanoparticle-related. Atelectasis and emphysema-like lesions are alterations related to lung inflation, so they can be considered an artifact related to either animal euthanasia or sample processing.

## 3. Discussion

Since, nanostructures reveal different features when passing through the organism, compared to substances in bulk or ionic forms, they become a promising tool to improve bioavailability of deficient substances. It is important to remember that comparing results between similar studies might be confusing, because nanoparticle physicality (size, shape, suspension etc.), route of administration and dose may strongly affect their behavior in the organism. Our previous studies reported efficient absorption of orally administered, zinc-based nanoparticles [33,34,35,36]. Hilty et al. also reported that nanosized Fe/Zn compounds presented higher bioavailability, compared to standard FeSO_4_ [40]. Moreover, verified in our studies, the oral route of nanoparticle administration prevented potential undesirable/toxic effects related with intravenous/intramuscular application of iron-doped substances [16,17,18]. Obtained results indicated the efficient and rapid transfer of orally administered ZnO:Fe^3+,2+^ NPs to hematopoietic tissues—spleen and bone (which included bone marrow)—with an increase in iron level detected in those tissues as soon as 24 h following even a single application of nanoparticles (Figure 9b,c). These findings are inconsistent with similar studies, where spleen and liver were indicated as main target organs [29,30,31,32,41,42], however aforementioned peculiarities of nanomaterials must be acknowledged. In the cited studies the iron accumulation was revealed mostly 1–2 days post iron-based nanoparticle administration, most probably a result of nanoparticle sequestration by mononuclear macrophages, which carry accumulated nanostructures to the tissues of liver or spleen. Thereafter, iron released in both spleen and liver resulted in a rapid increase of reported serum iron [29,30]. Those studies showed different organism circulation mechanism of nanomaterials, which overwhelmed natural iron homeostasis pathways, this may lead to excess iron accumulation and in consequence to the toxic effects or disruption of natural iron circulation mechanisms. Furthermore, iron-based nanostructures overcoming the blood-brain barrier has also been previously reported [29,30,31,32], even followed by neurological disturbances [43,44]. In contrast, our study showed unchanged iron levels in the brain, heart, skeletal muscle and lung tissue, as well as unchanged level of serum iron which suggests safety of manufactured material and a rather slow biodegradation of nanoparticle matrix with gradual release of iron ions (Figure 9h,i). Increased iron level in kidneys 24 h after application of ZnO:Fe^3+,2+^ NPs (Figure 9d), might be related with primary renal excretion phase, which was previously noted specifically for fractions of smaller (<50 nm) nanoparticles [45]. Increased levels of iron, observed in the small intestine, are predominantly associated with our preferred oral delivery route and thus were expected. Furthermore, our NPs seem to be better adapted to the oral route of administration, as no significant stomach dissolution of the matrix was previously reported [35]. In his paper, Hilty et al. did not observe any iron deposition in the small intestine, which was explained by complete dissolution of nanomaterial in the stomach, associated with the premature release of iron dopant [40]. The increase in iron levels in skeletal muscle and kidneys, observed in our study in the control group (ZnO nanoparticles without iron doping), requires further evaluation, as at this stage it cannot be explained.

Elevated blood/serum iron level was observed in literature studies mostly at the early stage, 6 h following intragastric application of Fe_3_O_4_ nanoparticles [29] and within a few hours after intravenous injection of iron oxide nanoparticles [42]. Those studies cannot be directly compared with our model, as those nanoparticles are non-biodegradable. Hence, observed iron deposition should be attributed to physical process of NPs deposition on/in the erythrocytes and not the increase in the bioavailable iron pool in the organism. Moreover, in our study, the shortest experimental time-point following nanoparticle administration was set at 24 h, therefore no elevated serum iron concentration in the experimental groups could be justified (Figure 8 and Figure 12). On the other hand, we showed increased level of hemoglobin, RBC and hematocrit parameters, mostly 24 h or 1 w following single application of ZnO:Fe^3+,2+^ nanoparticles (Figure 8a,b,f). According to other authors, elevated RBC (and consequently hemoglobin) level following intravenous injection of iron oxide nanoparticles was related with additional oxygen requirement for nanoparticle distribution and catabolizm [42]. Stankovic et al. observed erythrocyte and hematocrit increase in rats with induced acute inflammation [46], which may also explain aforementioned observation via the increase of the oxidative stress and inflammatory parameters. Similarly, decreased levels of mean corpuscular hemoglobin (MCH) and mean corpuscular hemoglobin concentration (MCHC) parameters might also be the effect of inflammatory processes induced by acute/chronic application of ZnO:Fe^3+,2+^ nanoparticles (Figure 12c,d). However, this seems unlikely, as no changes in pathomorphological parameters were observed in any experimental groups. Increased levels of red blood cells (trend), hemoglobin and hematocrit were observed 72 h after acute and 1 w after chronic model of ZnO:Fe^3+,2+^ application. These most likely indicate that iron-doped nanoparticles were able to reach hemopoietic tissues and improve hematopoiesis by gradual iron release from nanoparticle matrix (Figure 12a,b,f).

Defining the distribution pattern of orally given iron-doped nanostructures to living organism seems to be crucial for safe usage of the nanomaterial. Some authors concluded [47,48], that in rats, orally delivered nanoparticulated Fe^3+^ undergo the standard, duodenum absorption with further (ferroportin-depended) efflux from enterocytes. Authors also revealed similar organism reaction to exogenous nanoparticulated Fe^3+^ and standard FeSO_4_ supplementation [47]. Similarly, Rohner et al. did not report any significant changes in bioavailability between standard and nanoparticulate FeSO_4_. Authors, considering the high solubility of nanoparticulate FeSO_4_, also suggested the absorption through typical, duodenal iron pathways [48]. Our previous study showed that ZnO nanoparticles remain stable in the stomach juice and only a relatively small portion of the matrix is dissolved [35]. The main event of gradual dissolution happens at the tissues targeted by nanoparticles [34,35]. Hence we have chosen this matrix for our trial at iron supplementation strategy.

One of the most important parts of standard iron regulation mechanism is hepcidin. This hormone is mostly secreted by the liver and negatively regulates iron absorption and its release from macrophages, generally shutting the iron trans-organ circulation and homeostasis. Increased expression of hepcidin is usually induced by elevated iron levels in the organism, but the hormone also plays an important role as a part of organism-defense system throughout pathological conditions [49,50,51]. Prolonged infection, chronic inflammation or certain diseases (e.g., chronic kidney disease) result in high level of circulating hepcidin and consequently anemia. Current, approved iron supplementation strategies, also acutely increase hepcidin levels, which was postulated a further complication for anemic organism [9,23]. Thus, novel iron delivery systems, which avoid activation of hepcidin expression seem to be crucial for success of supplementation strategy. The results of the current study show no effect of orally administered nanoparticles based on ZnO matrix, on the expression of hepcidin (Figure 10 and Figure 13). This may indicate that our supplementation strategy either avoids the regulatory pathways of hepcidin, or that iron is gradually released from the matrix, preventing accumulation of high quantities of free ions in the circulation. Similarly, other study showed that even though iron nanoparticles increased hepcidin expression, reported levels were greatly lower when compared to the standard supplementation [52]. On the other hand, findings of Liskova et al. showed increased hepcidin expression following intravenous application of iron nanoparticles to rats [53], which may be a result of the non-physiological administration route. We could attribute it to increased erythrocyte catabolism, rather than the effect of delivered iron.

It is widely known that delivery of exogenous iron can lead to toxic side effects, e.g., production of harmful free radicals (ROS) [18]. Similarly, in other studies rats were given acute/chronic oral doses of iron oxide material in nano/bulk form and various biochemical enzyme activities in differed organs were assessed [43,44]. Those results showed that iron-oxide nanoparticles induced neurological and physiological disturbances, along with affecting the metabolic pathways of neurotransmitters, the cell-membrane permeability or causing liver and kidney necrosis [43,44]. Interestingly, iron oxide in bulk form did not induce significant changes in biochemical parameters/histopathology in either study [43,44], which emphasized the importance of size in the toxicity of nanomaterials. To assess potential nanotoxicity of ZnO:Fe^3+,2+^ nanoparticles, used in the current study, the level of hepatic activity of glutathione peroxidase was used as marker of iron-induced oxidative stress following acute/chronic exposure simulation. Results showed non-statistical changes in the levels of antioxidant enzyme (GPx) after administration of nanoparticles in all tested groups (Figure 13a), despite an increase observed 24 h following acute simulation.

Ferritin is a protein responsible for iron storage in the living organism, as well as for the prevention against toxic iron overload [20]. It has been confirmed that iron-based nanomaterials are able to transform (degrade) from exogenous nanoparticles to endogenous iron storage, increasing the levels of ferritin proteins within liver and spleen [54]. Our study revealed statistically elevated level of L-ferritin in the liver and spleen at 1 w and 1 m following single/chronic application of iron-doped ZnO NPs (Figure 11). This could indicate the gradual release of iron ions from the nanoparticle matrix. Another study [55] showed a peak of ferritin at 24 h post intravenous administration of magnetic iron-based nanostructures. Theoretically, this could contradict our observations, yet, as previously [53] it was most likely linked with the administration route. Interestingly, no ferritin increase (neither in spleen, nor in the liver) was observed in the current study following acute application model of iron-doped ZnO nanoparticles. On the other hand, 1 w after chronic application model of nanoparticle delivery, liver ferritin level was statistically elevated (Figure 14).

Therefore, the ferritin increase, along with improved, crucial hematological parameters (RGB, HGB, HCT) and lack of the influence on hepcidin expression, indicate the chronic application (multiple dose, with longer intervals) of iron-doped nanostructures to be the most effective among tested.

## 4. Materials and Methods

### 4.1. Preparation of Nanoparticles

ZnO:Fe nanoparticles were prepared using microwave hydrothermal technique [33,34,35,36]. Two starting compounds as the iron ions source were used to obtain two kinds of samples: ZnO:Fe containing only Fe^3+^ ions and ZnO:Fe^3+^ enriched with Fe^2+^ ions. Mother liquid was aqueous solution of metals’ compounds. Solution was prepared by dissolving 12 g of zinc nitrate(V), Zn(NO_3_)_2_·6H_2_O (Sigma Aldrich 98%, Darmstadt, Germany) and 0.078 g iron(III) nitrate(V), Fe(NO_3_)_3_·9H_2_O (Sigma Aldrich 99.95%) in distilled water for sample with Fe^3+^ ions. Sample containing both Fe^2+^ and Fe^3+^ was prepared using aqueous solution of 14 g zinc nitrate(V), Zn(NO_3_)_2_·6H_2_O (Sigma Aldrich 98%) and 3 g iron(II) acetate, Fe(CO_2_CH_3_)_2_ (Sigma Aldrich 99.99%). Solution was alkalized with 5 m aqueous NaOH solution to pH = 12. Resulting residue was triply washed with distilled water, suction filtered and placed in teflon vessel. Volume of the vessel was 100 mL and it was filled with reaction mixture to 80%. Container was placed in Ertec Magnum II reactor operating at 2.45 MHz with power of 0.7 kW. Reaction was conducted by 20 min in the pressure of 6 MPa. After cooling down the reactor, resulting powder was dried overnight at the temperature of 40 °C. Product was ground in agate mortar to powder and afterwards analyzed. ZnO:Fe sample containing only Fe^3+^ ions was red colored and with additional Fe^2+^ was brownish green.

### 4.2. Characterization of Nanoparticles

X-ray diffraction (XRD) measurements were conducted with Phillips X’Pert powder diffractometer operating with CuKα radiation (0.154 nm). The measurements were conducted in the 2θ range from 10 to 110 with a step of 0.05 and at counting time of 3 s. Samples were dispersed in ethanol, dropped into the chamber and dried. Scanning electron microscopy (SEM) measurements were conducted with high resolution (1 nm) Hitachi SU-70 microscope, equipped with characteristic radiation detector (EDX) and cathodoluminescence system GATAN Mono CL3. Dry samples were dispersed in reverse osmosis (RO) water using Sonics VCX500 ultrasonic processor for 3 min, in 10 s pulses, then dropped on (111) Si crystal. Then they were dried at room temperature. Photoluminescence emission (PL) and excitation (PLE) spectra were taken using Horiba/Jobin-Yvon Fluorolog-3 spectrofluorometer, equipped with a xenon lamp as excitation source and Hamamatsu R928P photomultiplier. Samples were pressed into the solid sample holder and then placed into measurements chamber. X-ray photoelectron spectroscopy (XPS) measurements were performed with Scienta 4000 analyzer and monochromatic Kα Al radiation, for Zn and O lines and twin anode for wide scan and Fe 3p line to maintain reasonable intensity. Samples were measured in the dry form without sonication step. Powder was spread over carbon tape, therefore C lines were not analyzed quantitatively. Dynamic light scattering and zeta potential were measured with DelsaMax Pro particle characterization system (Beckman Coulter, Sp. z o.o., Warsaw, Poland). Samples were dispersed in RO water using Sonics VCX500 ultrasonic processor for 3 min in 10 s pulses. Then they were injected into 180 µL flow cell and placed in the holder. A 50 mW 532 nm DPSS laser diode was used for the measurement. Measurement was conducted in 10 cycles at 25 °C.

Nanoparticles size distribution was evaluated from SEM images—200 crystals were measured lengthwise and across. Dimension distribution was confirmed by DLS technique. Samples of 1 mg/mL RO water suspension were prepared using sonication (500 W ultrasound disruptor (Sonics, Newtown, CT, USA)) and analyzed.

### 4.3. Experimental Design, Animal Model and Sample Collection

The experiment was performed on adult, 3–6 month old, male Balb-c mice (*n* = 73). All animals were group-housed and kept under controlled living conditions: 25 °C, humidity 30%, 12 h day-night cycle (UniProtect Air Flow Cabinet, Merazet SA, Poznan, Poland). Standard feed and water were provided ad libitum. All experiments performed within current study were conducted in accordance with Local Ethical Committee (number of permission: WAW2/59/2017). During the study animals from experimental groups received, by oral gavage, 0.3 mL suspension of nanoparticles (10 mg/mL) in RO water. The control group did not receive any treatment. The whole experiment was divided into two parts (Figure 7). The main goal of the first part was to select better form of ZnO nanoparticles doping (only Fe^3+^ iron or a mixture of Fe^3+^ and Fe^2+^ iron). For this purpose, animals were divided into following groups based on administered nanoparticles: control group (CTRL), ZnO (ZnO nanoparticles without doping), Fe^3+,2+^ which received ZnO nanoparticles doped with both Fe^3+^/Fe^2+^ and Fe^3+^ group which received ZnO nanoparticles doped only with Fe^3+^. Following 24 h, 1 w (week) or 1 m (month) animals were sacrificed using CO_2_-O_2_ chamber (CO_2_ Box, Bioscape, Merazet) with EU-approved gas mixture and tissue, and blood samples were collected for analyses. The second part of the study was conducted with ZnO nanoparticles doped with Fe^3+^/Fe^2+^, selected from the first part of the study. Animals were divided into two groups: simulation of acute (Acute) and chronic (Chronic) exposure to the nanoparticles. Mice from the Acute groups received three doses of nanoparticles at 8 h intervals. Animals from the Chronic groups also received three doses of nanoparticles, but at 72 h intervals. Following the last administration, mice were sacrificed at 24 h, 72 h or 1 w with subsequent collection of tissues and blood samples.

Blood was drawn from mice by direct cardiac puncture instantly after death into heparin-coated tubes. Collected blood was divided for hematological and plasma analyses. Hematological evaluation was performed directly after blood collection. Samples for plasma analyses were centrifuged (4000× *g*, 7 min, 4 °C) for plasma separation, aliquoted and stored at −80 °C. Freshly collected organs were portioned and frozen in −20 °C (for Atomic Absorption Spectrometry analyses) or frozen in liquid nitrogen and stored at −80 °C for molecular analyses. Part of obtained tissues were fixed in Bouin’s solution (Sigma Aldrich) and following 24 h transferred to 70% ethanol. Afterwards, organs were embedded in paraffin, cut to 4 μm-thin sections and stained with hematoxylin and eosin (HE) for histopathological evaluation.

### 4.4. Hematological Parameters

Analyses of erythrocyte count (RBC), level of hemoglobin (HGB), hematocrit (HCT), mean corpuscular volume (MCV), mean corpuscular hemoglobin (MCH), mean corpuscular hemoglobin concentration (MCHC) and red cell distribution width (RDW) were conducted with automatic hematology analyzer (MINDRAY BC-2800vet).

### 4.5. Plasma Ferritin and Iron Measurement

Quantitative detection of plasma ferritin in mice was performed with a commercial kit (EM1019, Wuhan Fine Biotech Co., Wuhan, China), based on sandwich enzyme-linked immune-sorbent assay technology. The assay was performed according to the manufacturer’s instruction. Iron concentration in plasma samples was determined by colorimetric measurement of an iron-chromazurol complex, according to the manufacturer’s instruction (Biomaxima SA, Lublin, Poland).

### 4.6. Evaluation of Iron Level in Tissues

Quantitative measurements of iron concentration in collected tissue samples were determined with Atomic Absorption Spectrometry (AAS) method, described in detail previously [35]. The protocol requires weighing each sample and leaving for >16 h in the solution of 1 mL of 30% hydrogen peroxide and 5 mL of 65% nitric acid (Merck Sp. z o.o., Warsaw, Poland). Then, all samples were mineralized in the microwave system Ethos 900 (Milestone, Sorisole, Italy). After cooling down, iron content in liquid samples was quantified with AAS (Perkin-Elmer, Waltham, MA, USA).

### 4.7. Hepatic Hepcidin mRNA Quantification

In order to assess the level of hepatic hepcidin mRNA, the Real-Time quantitative RT-PCR method was performed. For this purpose, the total RNA was isolated from approximately 20 mg of liver using Trizol reagent (Invitrogen, Waltham, MA, USA) according to the manufacturer’s protocol. Reverse transcription reactions were performed using the Transcriptor First Strand cDNA synthesis Kits (Roche, Basel, Switzerland) and 2 μg of total DNAse-treated RNA. Real-time quantitative PCR analysis was performed with the Light Cycler U96 Software (Roche Diagnostics) using gene-specific primer pairs (Table 3). The quantification of the mouse hepatic hepcidin mRNA was detected using SYBR Green I (Roche Diagnostics). Specificity of the amplicon was checked by subjecting the PCR products to melting curve analysis and further electrophoresis. For the data analysis the Light Cycler U96 Software (Roche Diagnostics) was used. Hepatic hepcidin mRNA levels were normalized to transcripts encoding hypoxanthine-guanine phosphoribosyltransferase genes (Hprt). Relative Hamp expression was determined using the −ΔCt method.

### 4.8. Immunoblot Analyses

For the detection of liver and spleen L-ferritin subunit, approximately 50 μg of respective tissue samples were homogenized in 300 μL buffer (0.25 mol/L sucrose, 0.03 mol/L histidine, pH 7.2), supplemented with cocktail of protease inhibitors (Sigma-Aldrich). The homogenates were centrifuged (6000× *g*, 15 min, 4 °C) followed by ultracentrifugation of supernatant (80,000× *g*, 45 min, 4 °C). Protein concentrations were determined by the Bradford assay (Biorad, Hercules, CA, USA). Afterwards, obtained liver/spleen cytosolic protein extracts were resolved by electrophoresis on 14% SDS-polyacrylamide gels. Proteins were transferred from the gels to PVDF membranes (Thermo Scientific, Waltham, MA, USA). Then membranes were blocked with 7% skimmed milk for 2 h at room temperature. Subsequently, an overnight incubation at 4 °C with the primary antibodies against recombinant mouse light chain ferritin (L-Ft, a gift from Dr. Paolo Santambrogio, Milano, Italy) were performed. Following washing, membranes were incubated (1 h at room temperature) with secondary horseradish peroxidase (HRP)-labeled antibodies (goat anti-rabbit, polyclonal, Sigma-Aldrich) for chemiluminescence detection. Quantities of protein were assessed by scanning densitometry using Quantity One software (Biorad).

### 4.9. Analysis of Glutathione Peroxidase (GPX) Activity

Hepatic activity of glutathione peroxidase was measured with a colorimetric commercial kit (ab102530, Abcam, Cambridge, UK), according to the manufacturer’s instruction. For sample preparation liver was washed in PBS, resuspended in assay buffer, homogenized and centrifuged (12,000× *g*, 15 min, 4 °C).

### 4.10. Histopathology

The collected biological samples of tissue samples (liver, kidney, spleen, brain, duodenum, lungs) were fixed for 24 h in Bouin’s solution (Sigma Aldrich, St. Louis, MO, USA), then cut and placed in standard plastic briquettes for tissue specimens. Then samples were rinsed in running water, dehydrated in grades of ethanol and xylene and embedded in paraffin. Paraffin blocks were cut in 4 µm slices and stained with hematoxylin-eosin (HE). Afterwards, slides were examined by a veterinarian pathologist.

### 4.11. Statistical Evaluation

Data presented in current paper are shown as the mean values ± SEM. For statistical evaluation of obtained results, the one-way ANOVA with Tukey’s post hoc tests were performed for comparison between control and experimental groups (Graphpad InStat 3.1, San Diego C, CA, USA). Statistical analyses and figures were prepared with Graphpad Prism 5. For all assessments, obtained data were considered significant at *p* ≤ 0.05, whereas *p* ≤ 0.01 and *p* ≤ 0.001 were considered as highly and extremely significant. Trend was established for *p* ≤ 0.1.

## 5. Conclusions

In conclusion the present study aimed at investigating the possibility of utilization of zinc oxide nanoparticles for iron delivery as oral supplementation strategy. We established that, doping the ZnO matrix with a mixture of Fe^3+^ and Fe^2+^ rather than plain Fe^3+^, seemed to be more effective from a physiological point of view. Taken together our results showed that the chronic supplementation model could be the most effective strategy, since we observed an improvement of crucial hematological parameters and the increase of ferritin without the activation of hepcidin expression.

## Figures and Tables

**Table 1:** Energy-dispersive X-ray spectroscopy (EDX) measured elemental composition of ZnO:Fe^3+^ and ZnO:Fe^3+,2+^ samples. Concentrations given in atomic %.

	Zn	O	Fe	N	Cl
ZnO:Fe^3+^	38.9 ± 0.5	54.7 ± 0.4	1.4 ± 0.1	5.1 ± 0.4	-
ZnO:Fe^3+,2+^	22.0 ± 0.4	65.8 ± 0.5	0.4 ± 0.0	11.6 ± 0.8	0.1 ± 0.0

**Table 2:** X-ray photoelectron spectroscopy (XPS) measured elemental composition of ZnO:Fe^3+^ and ZnO:Fe^3+,2+^ samples. Concentrations given in atomic %.

	Zn	O	Fe	N	Cl
ZnO:Fe^3+^	37.9	59.0	0.8	2.3	-
ZnO:Fe^3+,2+^	42.0	52.3	0.3	1.8	3.6

**Table 3:** Primers for RT-PCR.

Gene	Primer Sequence 5′-3′	
	Forward	Reverse
Hamp	TCTCCTGCTTCTCCTCCTTG	CAATGTCTGCCCTGCTTTCT
Hprt	AGGGAGAGCGTTGGGCTTAC	TCGCTAATCACGACGCTGGG

## Data Availability

Data is contained within the article.
